# Influence of Natural Wollastonite Microfibers on the Mechanical Behavior of Ultra-High-Toughness Cementitious Composites Containing Polyethylene Fibers

**DOI:** 10.3390/ma19091717

**Published:** 2026-04-23

**Authors:** Shujuan Wang, Guanjie Li, Feng Luo

**Affiliations:** 1 School of Intelligent Construction, Jilin University of Architecture and Technology, Changchun 130111, China; 2 Changchun City Urban Construction Maintenance Group Co., Ltd., Changchun 130021, China; 18686518721@163.com; 3 Key Laboratory of Automobile Materials of Ministry of Education, Department of Materials Science and Engineering, Jilin University, Changchun 130025, China; luofeng@jlu.edu.cn

**Keywords:** wollastonite, flexural strength, PE fibers, fluidity, compressive strength, tensile properties

## Abstract

**Highlights:**

- This paper investigates the effects of partially replacing cement with wollastonite inorganic microfibers on the fluidity, flexural strength, and compressive strength of ultra-high-toughness cementitious composites containing polyethylene (PE) organic fibers.
- This paper focuses on the influence of replacing cement with wollastonite microfibers of different aspect ratios on the initial cracking tensile strength, ultimate tensile strength, and tensile strain of ultra-high-toughness cementitious composites under uniaxial tension.
- Based on mechanical test results, observations of microstructures, and crack patterns, combined with the strain-hardening multiple cracking model of UHTCCs, the changes in the performance relationships among PE fibers, matrix, and PE fiber/matrix interfacial bonding after replacing cement with wollastonite microfibers are analyzed.
- The mechanism regarding the effect of cement replacement by wollastonite microfibers on the properties of ultra-high-toughness cementitious composites is elucidated.

**What are the main findings?**

- When the amount of cement replaced by wollastonite exceeds 4%, it exerts an adverse effect on the fluidity of UHTCCs. Compared with a small aspect ratio of wollastonite microfibers, a larger aspect ratio of wollastonite microfibers has a more significant negative impact.
- Wollastonite has a more obvious enhancing effect on the flexural strength and compressive strength of UHTCCs. However, the strength-enhancing effect of wollastonite with a larger aspect ratio begins to decrease when the replacement amount exceeds 6%.
- The addition of wollastonite not only increases the initial cracking tensile strength and ultimate tensile strength, but also increases the ultimate tensile strain of UHTCCs, and wollastonite microfibers with a larger aspect ratio exhibiting a more significant reinforcing effect.
- Replacing a part of the cement with wollastonite microfibers reduces defects in the matrix, enhances matrix strength, and improves the interfacial bonding performance between PE fibers and the matrix. It optimizes the performance relationship among PE fibers, matrix, and PE fiber–matrix interface and strengthens the synergistic effect of the three components.

**What are the implications of the main findings?**

- This paper can provide guidance for the application of wollastonite in ultra-high-toughness cement-based materials.

**Abstract:**

Wollastonite is a natural meta-silicate mineral material with fibrous characteristics. In this paper, wollastonite with different aspect ratios obtained after grinding was used as a mineral admixture to replace cement for preparing ultra-high-toughness cement-based composites (UHTCCs). The effects of wollastonite on the fluidity, compressive strength, flexural strength, and tensile properties of UHTCCs were investigated, and the crack morphology and micro-topography of the tensile specimens after fracture were observed. The experimental results show that when the wollastonite replacement ratio exceeds 4%, it exerts a negative effect on the fluidity of UHTCCs, and wollastonite with a larger aspect ratio has a more significant negative impact. Relying on the bridging effect, replacing cement with wollastonite can significantly improve the flexural strength and compressive strength of UHTCCs. However, when the replacement ratio exceeds 6%, the strength enhancement effect of wollastonite with a larger aspect ratio begins to decrease. When the cement replacement ratio of wollastonite is up to 6%, it can increase the initial cracking strength, tensile strength and tensile strain of UHTCCs. At the same replacement ratio, wollastonite with a larger aspect ratio shows a better reinforcing effect. According to the observation of post-fracture crack morphology, the cracks of UHTCCs change from the original smooth cracks to tortuous ones after cement is partially replaced by wollastonite. Replacing a part of cement with wollastonite optimizes the performance relationship among PE fibers, the matrix, and the PE fiber–matrix interface, and it enhances their synergistic effect. This not only raises the initial tensile cracking strength of UHTCCs but also improves its tensile strain. In particular, wollastonite with a larger aspect ratio exhibits a more pronounced reinforcing effect.

## 1. Introduction

Fracture brittleness is a typical characteristic of concrete using cement as the binder [1]. Fiber addition can significantly improve the toughness of cement-based materials. Ultra-high-toughness cementitious composites (UHTCCs) were developed precisely on the basis of fiber-reinforced cementitious materials, characterized by obvious tensile strain-hardening and stable multiple-cracking behaviors [2]. UHTCCs are designed according to the theories of micromechanics and fracture mechanics, and features high toughness, high ductility, and strong deformation resistance [3]. The ultra-high ductility and excellent tensile strain-hardening performance of UHTCCs are mainly realized through the steady-state crack propagation under the bridging effect between fibers and the cement matrix [4,5]. When subjected to bending or tensile loads, this material exhibits strong deformation resistance. The maximum crack width of UHTCCs can be controlled within 100 μm, and its tensile strain can exceed 3% [6].

Over the past few decades, the research and development of UHTCCs have advanced rapidly. Although many factors affect the tensile properties and multiple-cracking behavior of UHTCC, the type and performance of the fiber materials employed are the most critical. At present, the commonly used fibers mainly include polyethylene (PE) fibers [7,8] and polyvinyl alcohol (PVA) fibers [9,10,11,12]. Notably, UHTCCs prepared with PE and PVA fibers has already exhibited excellent tensile properties, with the maximum ultimate tensile strain exceeding 6%. In recent years, studies have indicated that when two or more types of fibers are used in cement-based composites, the reinforcing effect of hybrid fibers on the composites is better than that of a single fiber. In particular, UHTCCs prepared by hybridizing organic fibers with steel fibers not only has high deformability but also achieves high strength owing to the incorporation of steel fibers. For example, Ahmed et al. [8] developed an ultra-high performance concrete with strain-hardening behavior by mixing steel fibers of different lengths with PE fibers; this UHPC has a compressive strength of 150 MPa, a tensile strength of 15 MPa, and an ultimate tensile strain of 3%.

Wollastonite is a natural mineral material with an acicular crystal structure [13,14], and its aspect ratio generally ranges from 10 to 30 [15]. In recent years, research on preparing cement-based materials using wollastonite as an admixture has attracted increasing attention. According to Pawan Kalla [16], replacing 10–15% of cement with wollastonite can improve the compressive strength, flexural strength, and durability of concrete. This is because wollastonite can reduce porosity and densify the concrete microstructure. Relevant studies have also explored the application of wollastonite in ultra-high performance concrete (UHPC). Based on the exothermic curve of the cement-wollastonite mixture, wollastonite microfibers in UHPC can act as a passive internal restraint, leading to lower measured shrinkage strain. In addition, wollastonite microfibers can promote pore discontinuity, thereby reducing mass loss and drying shrinkage in UHPC [17]. Research results by Jianglin Zhu [18] indicate that wollastonite can enhance the mechanical properties of cement paste through bridging and pull-out effects. Rakibul I. Khan [19] studied the influence of ground wollastonite on cement hydration kinetics and strength development; the results showed that ground wollastonite accelerated the hydration of tricalcium silicate and the reaction of aluminate phases. Furthermore, wollastonite with a mean particle size of 3.5 μm consumed some calcium hydroxide via a slow pozzolanic reaction. Kuldasheva [20] investigated the effect of mineral wollastonite fibers on the flexural and compressive strength of Portland cement mortar; the results showed that incorporating wollastonite fibers into the cement system increased flexural strength by 132% and compressive strength by 140%. Dey [21] examined the improvement effects of various grades of wollastonite microfibers on the compressive strength and flexural performance of beam specimens. The results suggest that wollastonite microfibers moderately increase compressive strength, and significantly enhance fracture strength and toughness, leading to improved ductility and resistance to crack propagation.

Although research on the application of wollastonite in cement-based materials has been conducted for a relatively long period, most existing studies have focused on hydration heat, fluidity, compressive strength, shrinkage, durability, and other properties. Research on the effects of wollastonite on the fracture toughness and deformation performance of cement-based materials remains limited. Hasan Erhan Yücel [22] investigated the effects of two types of synthetic wollastonite with high aspect ratios on the rheological properties, strength characteristics, and flexural performance of engineered cementitious composites (ECC). The results indicated that wollastonite microfibers with an aspect ratio of 44:1 decreased the workability of ECC, which was attributed to the interlocking effect induced by the acicular particle morphology of wollastonite during flow. With an increase in the cement replacement level by wollastonite, the compressive strength of ECC declined while its ductility was improved. In comparison, when wollastonite was used to replace fly ash, both the compressive strength and flexural strength of ECC could be enhanced.

To date, research on the effects of wollastonite microfibers on the compressive and flexural strengths of cement-based materials is relatively mature. However, studies on their reinforcing effect on tensile properties under direct tension are limited. In particular, when wollastonite, as an inorganic fiber, is used together with PE organic fibers to prepare ultra-high-toughness cementitious composites (UHTCCs) for uniaxial tension tests, its reinforcing mechanism in cement-based materials remains unclear. The mechanical properties of UHTCCs are related to the matrix and the interfacial bond between fibers and the matrix. It is still unclear how the replacement of cement by wollastonite inorganic fibers affects the matrix strength and the interfacial bond performance between fibers and the matrix. In addition, it is not fully understood how wollastonite influences the initial cracking strength, tensile strength, tensile strain, crack width, and crack morphology of cement-based materials under uniaxial tension.

Therefore, in this study, two types of naturally ground wollastonite with different aspect ratios were selected as mineral admixtures to partially replace cement for preparing ultra-high-toughness cementitious composites (UHTCCs). The influence of wollastonite on the performance of UHTCCs was investigated, including fluidity, compressive strength, and flexural strength. In particular, this study focused on the effect of cement replacement by wollastonite on the tensile properties of UHTCCs containing PE organic fibers, such as initial cracking strength, tensile strength, and tensile strain. Based on macroscopic mechanical tests and microstructure and crack morphology observations, combined with the strain-hardening and multiple cracking model of UHTCCs, the changes in the performance relationships among PE fibers, the matrix, and the PE fiber–matrix interfacial bond after partial cement replacement by wollastonite microfibers were analyzed. The mechanism by which wollastonite affects the performance of UHTCCs was revealed.

## 2. Materials and Methods

### 2.1. Raw Materials and Mix Proportions

The raw materials used in this study included cement, silica sand, solid high-performance polycarboxylate superplasticizer (SP), wollastonite, polyethylene (PE) fibers, and water. The SP, with a water reduction rate of 35%, was supplied by Sobute New Materials Co., Ltd. (Nanjing, China). The cement used was ordinary Portland cement with a strength grade of 52.5 MPa, complying with the Chinese standard GB 175-2007 [23]. Silica sand with a particle size range of 198–400 μm was adopted.

**Figure 1:**
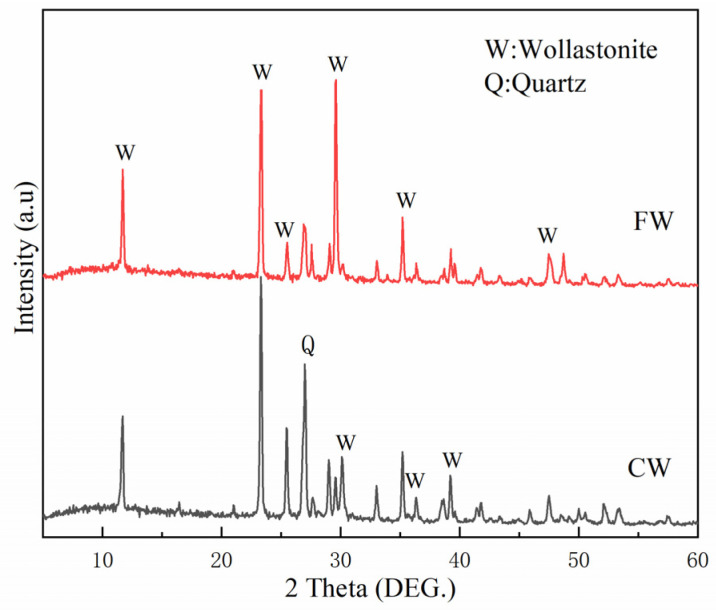
XRD patterns of FW and CW.

**Figure 2:**
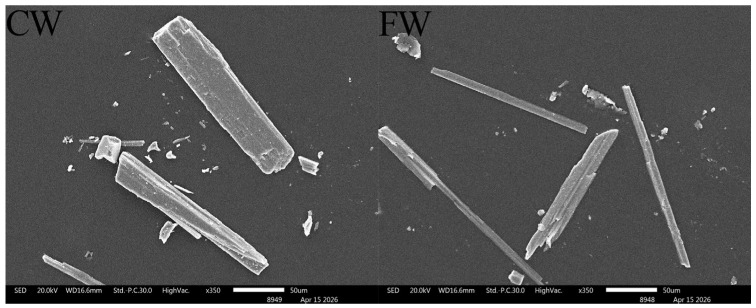
SEM images of CW and FW.

Wollastonite microfibers were supplied by Lishu Wollastonite Mining Company of China Nonmetallic Minerals Corporation (Siping, China). Two types of wollastonite with different aspect ratios were used in this experiment: the one with a relatively large aspect ratio had an aspect ratio of approximately 20–30 and a diameter of about 4–6 μm, while the one with a smaller aspect ratio had an aspect ratio of around 5–10 and a diameter of roughly 8–10 μm. Generally, wollastonite with a large aspect ratio has a finer particle size and is thus abbreviated as FW, whereas that with a small aspect ratio has a coarser particle size and is abbreviated as CW. The chemical compositions of cement, wollastonite, and silica sand are shown in Table 1. The XRD patterns and SEM images of wollastonite are presented in Figure 1 and Figure 2, respectively. The PE fibers had a length of 12 mm, a tensile strength of 3000 MPa, and an elastic modulus of 100 GPa.

The mix proportions of UHTCCs are listed in Table 2. PE fibers were incorporated at a volume fraction of 2.0%. UHTCC specimens were prepared by replacing 2%, 4%, 6%, 8%, and 10% of the cement by mass with each of the two wollastonite grades of different aspect ratios.

### 2.2. Specimen Preparation and Curing

UHTCCs were prepared as follows: cement, wollastonite, silica sand, and superplasticizer (SP) were dry-mixed in a planetary mixer for 3 min; water was then added, and mixing lasted another 3 min to obtain flowable fine mortar; subsequently, PE fibers were incorporated and mixed for 3 min to avoid fiber agglomeration.

**Figure 3:**
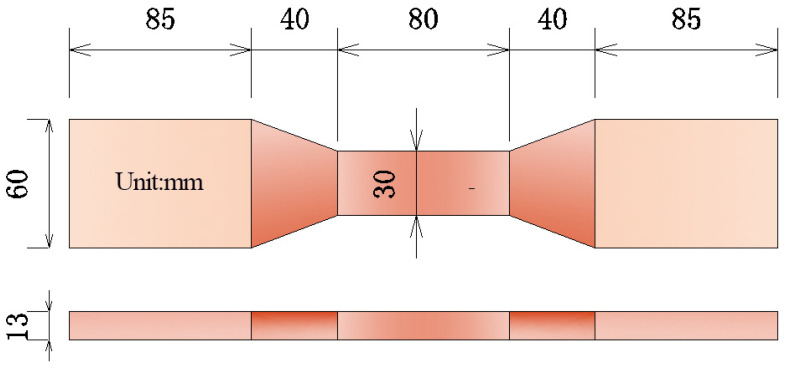
Dimension of direct tensile specimen.

The fresh mixture was poured into prefabricated molds. The specimens were demolded after 24 h and cured in a standard curing room at (20 ± 2) °C and relative humidity above 95% for 28 days. Prismatic samples (40 mm × 40 mm × 160 mm) were used for compressive and flexural tests, while dumbbell-shaped specimens were adopted for uniaxial tensile tests. Their detailed dimensions are shown in Figure 3.

### 2.3. Test Methods

The fluidity of fresh cement fine mortar was tested in accordance with the Chinese standard GB/T 2419-2005 [24]. Compressive and flexural strength tests of UHTCCs were carried out according to the Chinese standard GB/T 17671-2021 [25]. In line with this standard, three specimens per mixture were prepared for flexural strength testing, while on their halves the compressive strength was determined. The loading rate was 50 N/s for the flexural strength test and 2.4 kN/s for the compressive strength test. Tensile tests were performed at a loading rate of 0.5 mm/min and three specimens were tested per mixture. An extensometer was used to measure tensile strain over a gauge length of 50 mm. Microstructural observations of UHTCC specimens were conducted using scanning electron microscopy (JEOL, IT300, Japan Electron Optics Laboratory Co., Ltd., Tokyo, Japan). The SEM samples were extracted from the fracture surfaces of the tensile specimens after testing. All samples were gold-coated prior to observation. The mineralogical composition of raw materials was analyzed through X-ray diffraction (DX-2700 diffractometer, HaoYuan Instrument, Dandong, China) with Cu Kα radiation operated at 35 kV and 25 mA. The data were collected over a 2θ range from 5° to 60°, with a step size of 0.05°.

## 3. Results

### 3.1. Influence of Wollastonite on the Fluidity of UHTCC

**Figure 4:**
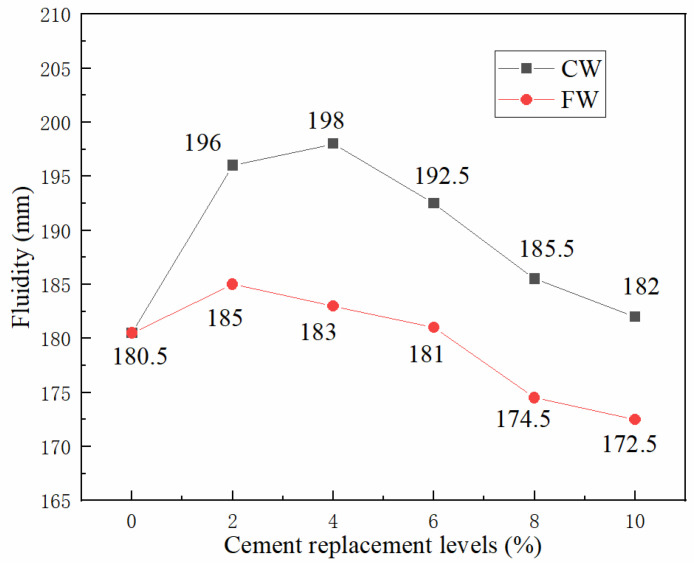
Influence of wollastonite on the fluidity of UHTCCs.

The influence of cement replacement with wollastonite of different aspect ratios on the fluidity of UHTCCs is illustrated in Figure 4. As shown in Figure 4, both types of wollastonite induced a first increase followed by a decrease in UHTCCs’ fluidity with the increase of cement replacement ratio, though slight differences were observed in the experimental data. For CW, the fluidity reached a maximum of 198 mm at a 4% cement replacement ratio, which was 9.7% higher than that of the blank sample (without wollastonite). With a further increase in the replacement ratio, the fluidity decreased gradually but remained higher than that of the blank sample even at the maximum replacement ratio of 10% set in this experiment. For FW, the fluidity peaked at 185 mm when the replacement ratio was 2%, which was only 2.5% higher than the value of the blank sample. A continuous increase in the replacement ratio led to a significant decline in fluidity. When the replacement ratio exceeded 6%, the fluidity fell below that of the blank sample. Overall, replacing cement with wollastonite improved the fluidity of UHTCCs when the replacement ratio was less than 4%, whereas it exerted an adverse effect when the ratio exceeded 4%. Compared with CW, FW had a more pronounced negative impact on the fluidity of UHTCCs.

Numerous studies have investigated the influence of wollastonite on the fluidity of cement-based materials, yielding inconsistent findings [13,26,27,28,29,30,31]. These discrepancies are primarily attributed to the aspect ratio and dosage of wollastonite employed [32]. The influence of wollastonite on fluidity is governed by two key factors: specific surface area and aspect ratio. On the one hand, wollastonite particles have a larger specific surface area than cement particles, which results in the adsorption of more free water and a consequent reduction in fluidity [19]. On the other hand, wollastonite has a high aspect ratio, and its acicular particles tend to form an interlocking network, thereby further reducing fluidity [33,34]. However, when the aspect ratio is within a low range, the acicular particles are less prone to interlocking; instead, they can dilute cement particles and break the flocculent structure formed during cement hydration, which is conducive to improving fluidity. The aforementioned experimental results indicate that FW has a higher aspect ratio, thereby exerting a more pronounced negative impact on fluidity, whereas CW with a lower aspect ratio is favorable for improving fluidity.

### 3.2. Influence of Wollastonite on the Flexural Strength of UHTCCs

**Figure 5:**
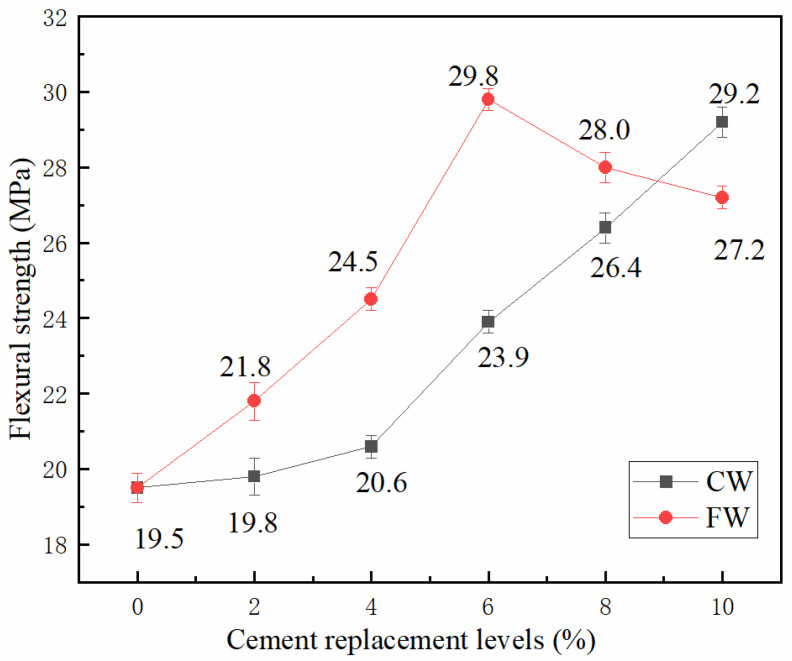
Influence of wollastonite on the flexural strength of UHTCC.

The influence of cement replacement by wollastonite with two different aspect ratios on the flexural strength of UHTCCs is presented in Figure 5. Within the replacement levels investigated in this study, both CW and FW contributed to the improvement of the flexural strength of UHTCCs.

For FW, the flexural strength reached a maximum of 29.8 MPa at a replacement ratio of 6%, representing an increase of 52.8% compared with the reference sample (19.5 MPa). With a further increase in the replacement ratio, the flexural strength started to decline. Nevertheless, even at a 10% replacement ratio, the flexural strength still reached 27.2 MPa, which was 39.5% higher than that of the reference sample. In comparison, the incorporation of CW had only a minor effect on the flexural strength of UHTCCs at replacement ratios below 4%, which differed noticeably from the behavior of FW. As the replacement ratio increased, the flexural strength of UHTCCs increased continuously. At the maximum replacement ratio of 10% adopted in this experiment, the flexural strength of UHTCCs reached 29.2 MPa, an increase of 49.7% relative to the reference sample. Overall, FW exhibited a more pronounced strengthening effect on the flexural strength of UHTCCs at low replacement ratios, whereas CW also achieved excellent performance in enhancing flexural strength at high replacement ratios.

Although inconsistent conclusions have been reported by different researchers [16,32,33,34], the influence mechanism of wollastonite on UHTCC performance can be interpreted using the “bridging effect” theory. First, the incorporation of wollastonite microfibers into cement-based materials exerts a filler effect, optimizing the pore structure of the cement matrix and enhancing its compactness. Second, fibrous wollastonite particles can impede crack propagation when the matrix is subjected to external loading [35].

**Figure 6:**
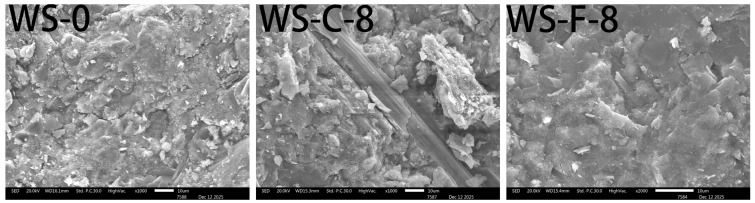
Micro-topography of the fracture surface of UHTCC specimens after flexural testing.

Figure 6 shows the micro-topography of the fracture surfaces of UHTCC specimens after flexural testing. It can be observed that UHTCCs present a denser microstructure when cement is partially replaced by wollastonite. During crack propagation, cracks extend through wollastonite particles, resulting in the fracture of fibrous wollastonite at the midsection. The fracture process of wollastonite microfibers dissipates a substantial amount of energy, which is macroscopically reflected as the improved flexural strength of UHTCCs. By comparing the micro-topographies of WS-C-8 and WS-F-8, it is found that WS-F-8 possesses a denser matrix structure. This is one of the key reasons why FW shows a more remarkable strengthening effect on the flexural strength of UHTCC.

In this experiment, FW exhibited a superior strengthening effect on flexural strength for two reasons. On the one hand, the smaller particle size of FW led to a more pronounced filler effect. On the other hand, FW possessed a larger aspect ratio, which enabled it to act more effectively as bridging fibers in addition to the filling contribution. Such bridging behavior restrained crack propagation during crack development, thereby giving rise to a better enhancement in flexural strength.

It should be noted that FW is not easy to disperse uniformly in the UHTCC matrix than CW, which leads to the enhancement effect of flexural strength reaching its peak earlier. When the cement replacement ratio exceeds the optimal value (6% for FW), the flexural strength begins to decrease significantly. This is because the poor dispersion of wollastonite with a larger aspect ratio causes local agglomeration in the matrix, affecting the strengthening effect on flexural strength.

### 3.3. Influence of Wollastonite on the Compressive Strength of UHTCC

**Figure 7:**
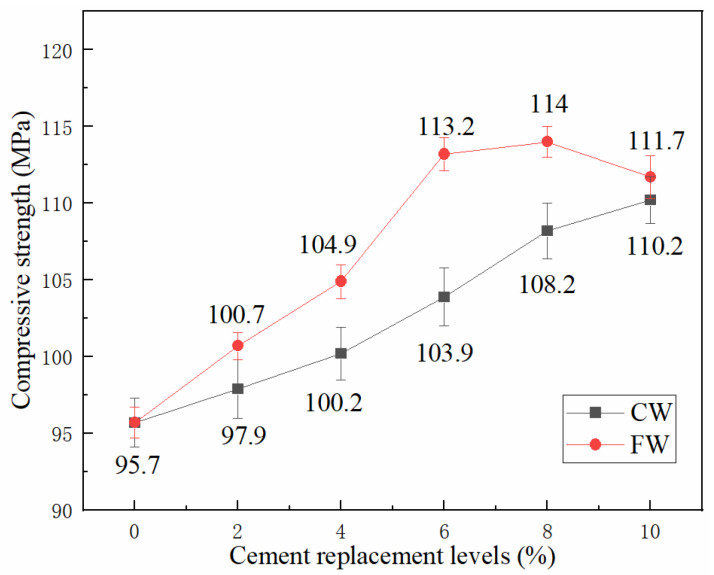
Influence of wollastonite on the compressive strength of UHTCC.

The 28-day compressive strength test results of the UHTCC samples incorporating different types of wollastonite are presented in Figure 7. As shown in Figure 7, replacing cement with two types of wollastonite with different aspect ratios exerted a positive influence on the compressive strength of UHTCCs. For FW, compared with the reference sample (without wollastonite), replacing 2%, 4%, 6%, 8%, and 10% of cement by mass, respectively, resulted in respective increases of 5.0%, 9.6%, 18.3%, 19.1%, and 16.7% in the compressive strength of UHTCCs. The compressive strength reached its maximum value when the replacement ratio was 8%. As for CW, in comparison with the reference sample, replacing 2%, 4%, 6%, 8%, and 10% of cement with CW led to increases of 2.3%, 4.7%, 8.6%, 13.1%, and 15.2% in the compressive strength of UHTCC, respectively. Within the replacement ratio range investigated in this experiment, the compressive strength of UHTCCs still showed an upward trend.

In addition, it can be observed from Figure 7 that at the same replacement ratio, FW exhibits a more pronounced strengthening effect on the compressive strength of UHTCCs than CW. Although FW has a larger aspect ratio and thus performs more effectively in inhibiting the propagation of microcracks, when the replacement ratio exceeds 8%, the compressive strength decreases, which is attributed to the poor dispersion of wollastonite with a larger aspect ratio. It causes local agglomeration in the matrix, affecting the strengthening effect on compressive strength.

### 3.4. Influence of Wollastonite on the Tensile Properties of UHTCC

**Figure 8:**
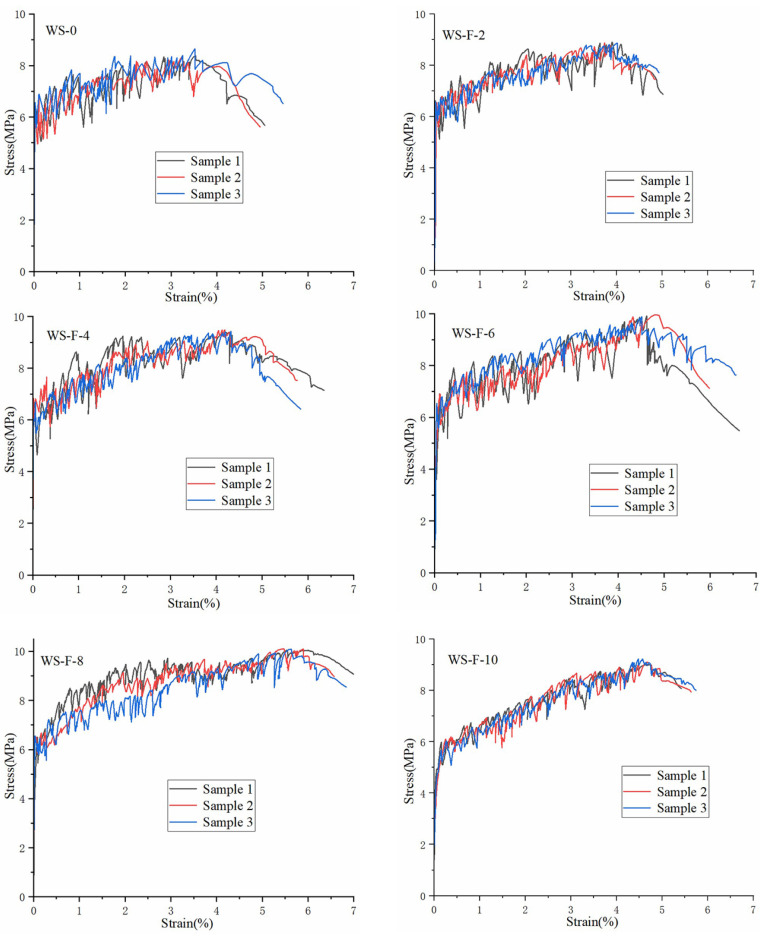
The tensile stress–strain curves of fine mortars containing FW.

**Figure 9:**
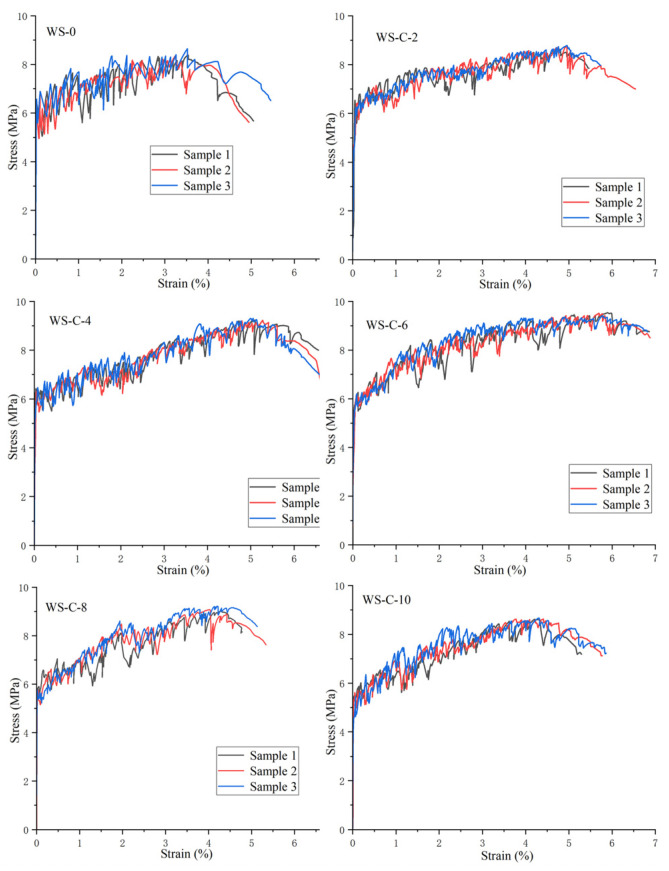
The tensile stress–strain curves of fine mortars containing CW.

This experiment investigated the tensile properties of UHTCCs after replacing cement with CW and FW, covering the first cracking strength, tensile strength, and tensile strain. By observing the fractured samples, the number of cracks and average crack width of the specimens were measured and calculated. Figure 8 and Figure 9 show the tensile stress–strain curves of UHTCCs with FW and CW replacing cement, respectively. Table 3 lists the measured first cracking strength, tensile strength, fracture strain, number of cracks, and average crack width. All specimens exhibited fluctuations in their stress–strain curves, which were caused by the matrix cracking and the bridging effect of fibers during the loading process—this is a typical characteristic of UHTCC.

From the stress–strain curves, it can be observed that the first cracking strength and tensile strength of the blank sample (without cement replacement) were 6.4 ± 0.3 MPa and 8.4 ± 0.2 MPa, respectively, with a final strain of approximately 3.3 ± 0.2%. When cement was replaced by FW, the first cracking strength, tensile strength, and strain of the UHTCCs all increased. When the replacement ratio of FW was 6%, the first cracking strength reached 6.7 ± 0.2 MPa. When the replacement ratio reached 8%, the tensile strength and strain peaked at 10.0 ± 0.1 MPa and 5.5 ± 0.1%, respectively. However, as the replacement ratio of FW exceeded 8%, the first cracking strength, tensile strength, and strain all showed a downward trend. In addition, the number of microcracks and average microcrack width of the samples were closely related to the replacement ratio of FW. With the increase in FW replacement ratio (up to 8%), the number of microcracks increased and the average crack width decreased. When the replacement ratio exceeded 8%, the number of microcracks decreased and the average crack width increased. This trend is consistent with the change rule of the mechanical properties of UHTCCs, which further confirms that the addition of wollastonite can effectively improve the tensile performance of UHTCCs.

When CW was used to replace cement, its influence on the first cracking strength, tensile strength, and fracture strain of UHTCCs exhibited a similar general trend to that of FW. However, the specific experimental results indicated that, at the same cement replacement ratio, CW exhibited a weaker enhancing effect than FW on the first cracking strength and tensile strength of UHTCCs. In contrast, CW outperformed FW in improving the strain of UHTCCs. For instance, at a 6% replacement ratio with CW, the first cracking strength, tensile strength, and strain were 6.2 ± 0.2 MPa, 9.5 ± 0.1 MPa, and 5.7 ± 0.1%, respectively. The number of microcracks was 46 ± 2, and the average microcrack width was 63.7 μm. In comparison, at a 6% replacement ratio with FW, the first cracking strength, tensile strength, and strain were 6.7 ± 0.2 MPa, 9.9 ± 0.1 MPa, and 4.6 ± 0.2%, respectively. The number of microcracks was 38 ± 2, and the average microcrack width was 60.8 μm.

### 3.5. The Surface Crack Morphology of Fractured UHTCC

**Figure 10:**
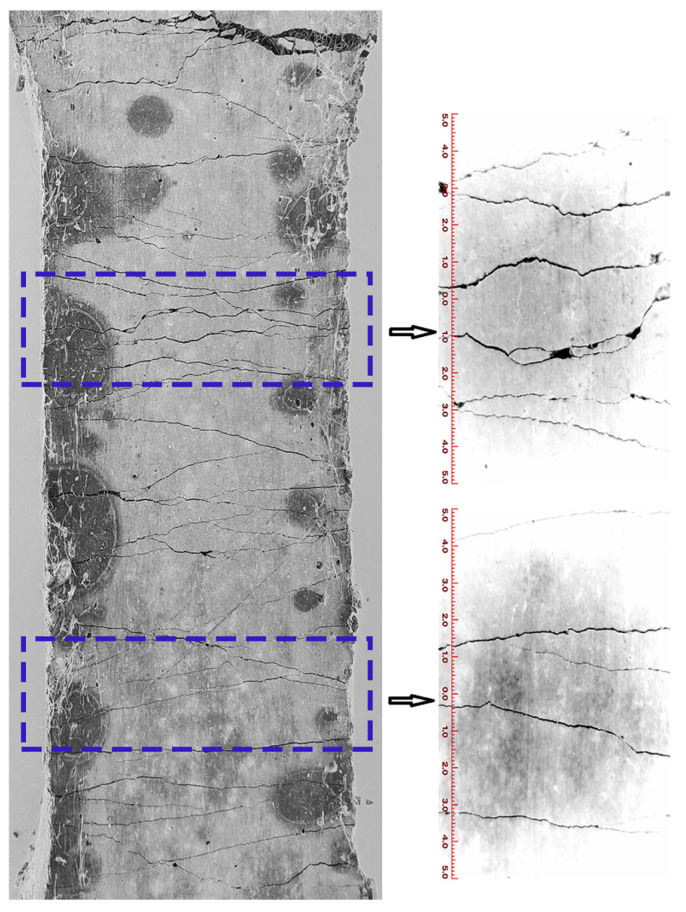
The surface crack morphology of WS-0.

**Figure 11:**
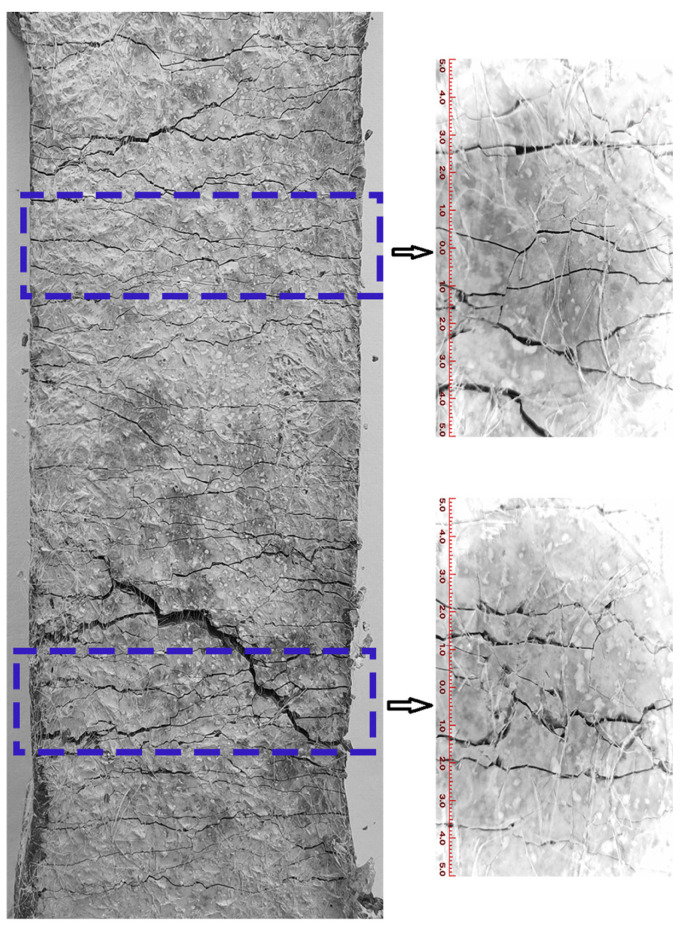
The surface crack morphology of WS-F-8.

**Figure 12:**
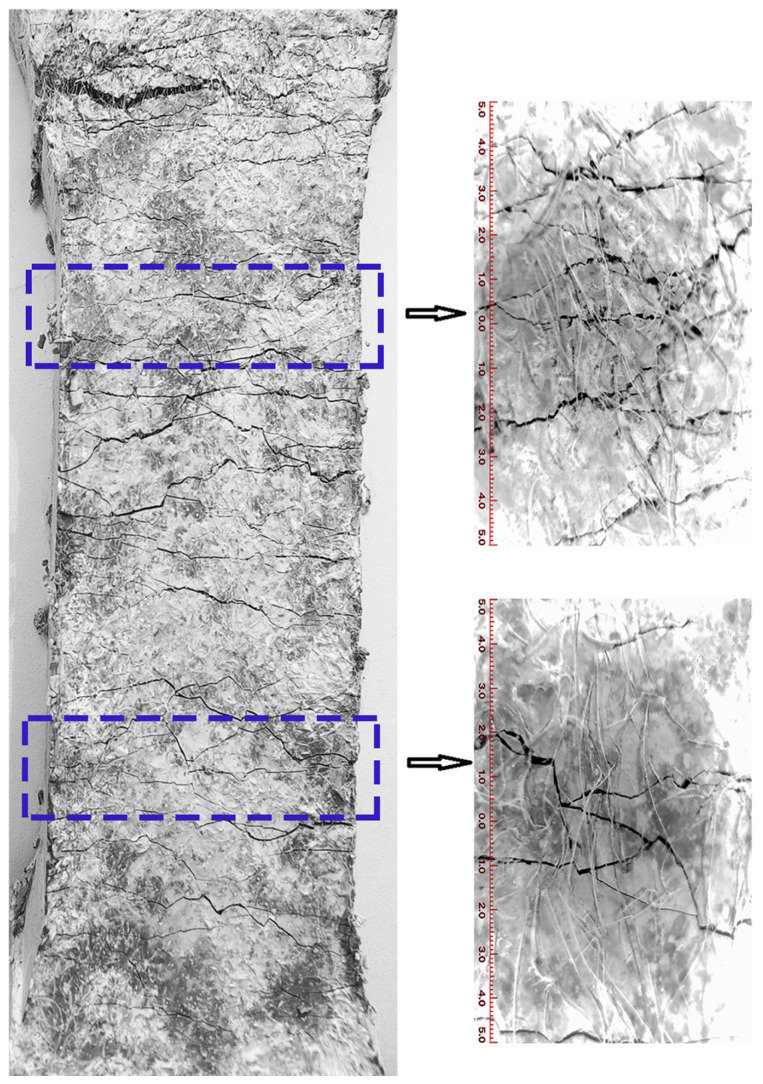
The surface crack morphology of WS-C-6.

The experiments were conducted to observe the surface crack morphology of three fractured UHTCC samples, namely WS-0, WS-F-8, and WS-C-6 (two of which exhibited the maximum tensile strain). The results are shown in Figure 10, Figure 11 and Figure 12. It can be observed from Figure 10, Figure 11 and Figure 12 that all three samples exhibited multi-crack propagation. In terms of crack morphology, most cracks in the blank sample (WS-0, shown in Figure 10) appeared smooth. After replacing cement with wollastonite in UHTCCs, the cracks in the samples (WS-F-8 and WS-C-6, shown in Figure 11 and Figure 12) became thinner and denser. Furthermore, from the perspective of crack propagation direction, the cracks showed varying degrees of deflection and intersection, forming a crisscross pattern both horizontally and vertically. Overall, the crack morphology of the samples with wollastonite addition was more tortuous compared to the blank sample.

### 3.6. Observation of Microscopic Morphology of Fracture Surfaces

**Figure 13:**
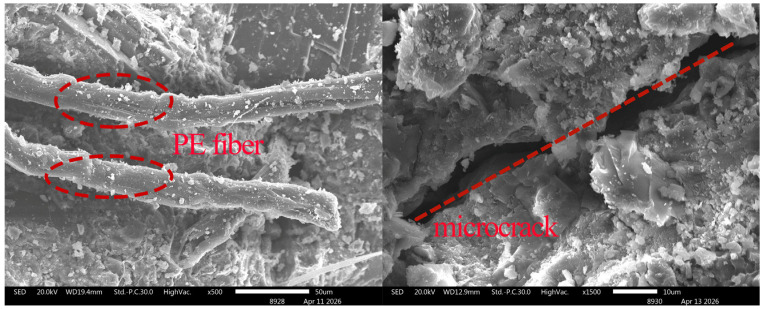
SEM imagines of fracture of WS-0.

**Figure 14:**
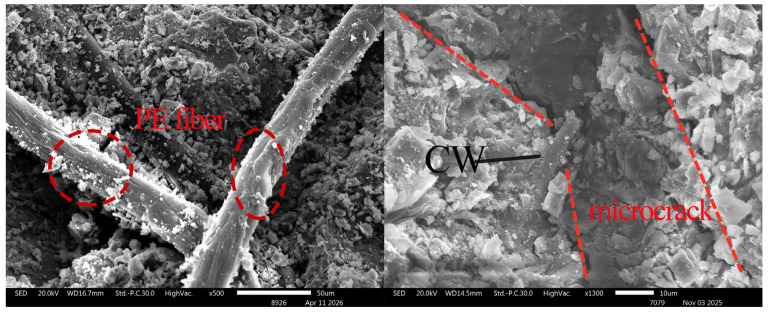
SEM imagines of fracture of WS-C-6.

**Figure 15:**
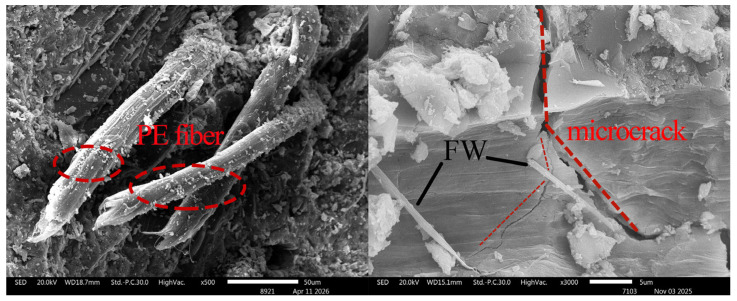
SEM imagines of fracture of WS-F-8.

The microscopic morphology of WS-0, WS-F-8, and WS-C-6 fracture surfaces after tension was observed using a scanning electron microscope (SEM), and the results are shown in Figure 13, Figure 14 and Figure 15. Key observations were focused on the pulled out PE fibers and microcrack regions in the experiments. Combined with the micro topographies shown in Figure 13, Figure 14 and Figure 15, it can be observed that obvious deflection occurred in the crack propagation direction in the regions where wollastonite particles were present. In Figure 14, acicular wollastonite exhibited a pull-out phenomenon at the crack locations, playing a role in bridging the cracks. Figure 15 clearly shows that in the regions with wollastonite, not only did the cracks deflect, but the width of the cracks passing through the wollastonite particles became narrower, indicating that wollastonite exerted an effect of inhibiting microcrack propagation.

From the perspective of the state of the pulled out fibers, obvious scratches were observed on the surface of the PE fibers in all three samples, which were caused by friction between the fibers and the matrix during the pull-out process. However, it can also be seen that compared with the PE fibers in the WS-0 sample, the fibers in the WS-C-6 and WS-F-8 samples had denser surface scratches and more attachments. This indicates that the addition of wollastonite microfibers in UHTCCs enhanced the interfacial bonding between the PE fibers and the matrix.

## 4. Discussion

In this study, UHTCCs were prepared by partially replacing cement with wollastonite. According to the experimental results reported in the literature [19], wollastonite exerts an influence on the hydration kinetics, phase formation, and compressive strength of cement due to the filler effect.

Both CW and FW contribute to the improvement of the compressive and flexural strengths of UHTCCs, which is associated with the filler effect and morphological effect of wollastonite microfibers. It is generally accepted that replacing cement with wollastonite microfibers enhances the compactness of cement-based materials, which is one of the mechanisms underlying the strengthening effect of wollastonite. Another mechanism is that wollastonite microfibers, as fibrous materials, can inhibit crack initiation and delay microcrack propagation. In this study, PE fibers and wollastonite microfibers exerted a synergistic effect on crack control, thereby enhancing the compressive and flexural strengths of UHTCCs. It is worth noting that as the cement replacement ratio by wollastonite increases, wollastonite microfibers with a larger aspect ratio reach their optimal replacement level more quickly. This may be attributed to the poor dispersibility of wollastonite microfibers with a larger aspect ratio, which are less likely to be uniformly dispersed in the matrix.

This study focuses on investigating the influence of cement replacement by wollastonite on the tensile properties of UHTCCs. The experimental results show that when the FW replacement ratio is within 6%, wollastonite can enhance the initial cracking strength, tensile strength, and fracture strain of UHTCCs.

**Figure 16:**
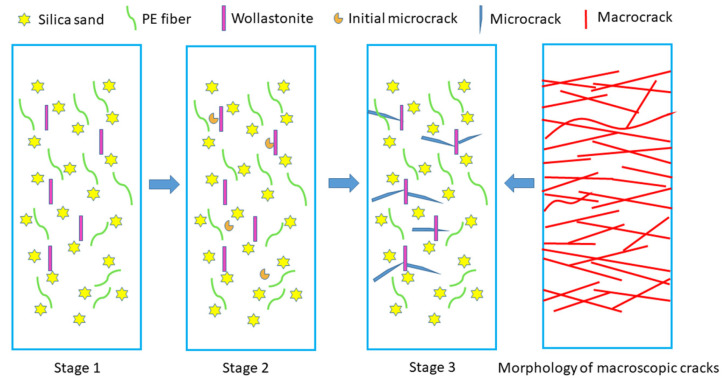
Schematic illustration of the influence of wollastonite microfibers on the tensile properties of UHTCCs.

Based on the failure mechanism of UHTCCs and the strain-hardening multiple cracking model, combined with the experimental results of crack morphology and fracture surface micro-topography of UHTCCs after tensile fracture, the action mechanism of wollastonite on UHTCCs under tensile stress can be explained using Figure 16. Specifically, wollastonite exerts its effect through two aspects. On the one hand, it improves the compactness of the matrix, thereby enhancing the initial cracking strength and tensile strength of UHTCCs. On the other hand, its needle-like structure can effectively inhibit the expansion of microcracks, reduce the crack width, and further improve the tensile toughness of UHTCCs.

(1). Unloaded stage

After replacing cement with wollastonite, although wollastonite does not participate in the hydration reaction of cement, its small particle size enables it to exert a filler effect in the hardened cement matrix, thereby improving the compactness of the matrix.

(2). Initial cracking stage

When UHTCC specimens are subjected to tensile loading, the first crack appears in the matrix once the tensile stress exceeds a certain value. The initial cracking tensile strength of UHTCCs is mainly related to the inherent defects within the material and the uneven fiber distribution.

The experimental results show that replacing cement with wollastonite microfibers increases the initial cracking tensile strength, indicating fewer defects in the matrix. The main reasons are as follows: on the one hand, wollastonite exerts a significant filler effect, which improves the compactness of the matrix; on the other hand, under tensile loading, fibrous wollastonite dissipates more energy during pull-out, thereby enhancing the initial cracking tensile strength.

It can be observed from the initial cracking strength test results that wollastonite microfibers with a larger aspect ratio exhibit a more pronounced improvement effect on the initial cracking tensile strength. This may be attributed to the larger bonding interface (in one direction per particle) between wollastonite microfibers with a higher aspect ratio and the matrix.

(3). Strain-hardening stage

When UHTCCs enter the strain-hardening stage, there are two key performance parameters: the ultimate tensile strength and the tensile strain. The strain-hardening behavior of UHTCCs is closely related to the properties of the fibers, the matrix, and the fiber/matrix interface. According to relevant theories of UHTCCs [36], the tensile strength of UHTCCs is mainly governed by two factors: the frictional bond and the chemical bond between the fibers and the matrix. PE fibers are organic fibers and have no chemical bond with the matrix. Therefore, the increase in tensile strength indicates an enhancement in the frictional bond between the fibers and the matrix. After partial replacement of cement with wollastonite, on the one hand, the frictional bond between PE fibers and the matrix is improved by modifying the microstructure of the matrix. On the other hand, when the PE fibers dispersed in the matrix are pulled out under tension, they may interact with surrounding wollastonite microfibers, which hinder the pull-out of PE fibers and dissipate more energy, thereby increasing the tensile strength of UHTCCs. Wollastonite microfibers with a larger aspect ratio have a higher probability of contacting PE fibers, which explains why their reinforcing effect is more significant.

The ultimate tensile strain of UHTCCs is mainly related to the matrix toughness and the interfacial bond performance between fibers and the matrix. According to the strain-hardening multiple cracking model proposed by Fisher and Li [36], a specimen must satisfy both the strength criterion and the energy criterion to achieve strain-hardening behavior. The strength criterion requires that the initial cracking stress of UHTCCs should be lower than the minimum bridging stress of the fibers (σ_fc_ < σ_o_, σ_fc_ is the initial cracking stress and σ_o_ is the minimum bridging stress of the fibers). However, an increase in matrix strength generally leads to a higher initial cracking stress, which is detrimental to the realization of strain-hardening.

The experimental results show that the addition of wollastonite enhances both the initial cracking strength and the ultimate tensile strain of UHTCCs. This indicates that replacing cement with wollastonite not only increases the initial cracking stress but also strengthens the bridging stress between the PE fibers and the matrix. In terms of matrix toughness, fibrous wollastonite exerts two roles when replacing cement. First, wollastonite provides a bridging effect that restricts the propagation of microcracks. Second, when microcracks encounter wollastonite particles, their propagation path deflects, preventing rapid crack growth along a single direction. Furthermore, as reflected in the improvement of tensile strength, replacing cement with wollastonite also enhances the interfacial bonding performance between PE fibers and the matrix.

In conclusion, experimental data demonstrate that partially replacing cement with wollastonite optimizes the synergistic relationship among PE fibers, the matrix, and the PE fiber–matrix interface, thereby enhancing their synergistic effect. This not only increases the initial tensile cracking strength of UHTCCs but also improves their tensile strain. In particular, wollastonite with a larger aspect ratio exhibits a more pronounced reinforcing effect.

## 5. Conclusions

This experiment explored the feasibility of preparing UHTCCs by replacing cement with wollastonite, and it compared the differences in the effects of wollastonite with different aspect ratios on the fluidity, compressive strength, flexural strength, and tensile properties of UHTCCs containing PE fibers. The main conclusions of the experiment are as follows:

1. When the wollastonite replacement ratio is less than 4%, it enhances the fluidity of UHTCCs; when the replacement ratio exceeds 4%, it exerts an adverse influence. Compared with CW, FW has a more pronounced negative impact on the fluidity of UHTCCs.
2. Wollastonite exhibits a significant enhancing effect on the flexural strength and compressive strength of UHTCCs. However, when the replacement ratio exceeds 6%, the strength-enhancing effect of FW begins to decrease. A possible reason for this is that FW is prone to causing particle agglomeration, thereby affecting the uniformity of the UHTCCs matrix and further influencing the strength enhancement effect and the stability of the material’s performance.
3. When the wollastonite replacement ratio does not exceed 8%, FW exerts a promoting effect on the first cracking strength, ultimate tensile strength, and fracture strain of UHTCCs. At the same replacement ratio, CW shows a weaker enhancing effect on the first cracking strength and ultimate tensile strength of UHTCCs compared with FW, but it has a higher fracture strain.
4. Observations on the microscopic morphology of UHTCCs fracture surfaces indicate that during the crack propagation stage, the propagation direction of cracks deflects when encountering wollastonite particles.
5. After replacing cement with wollastonite, the number of cracks in UHTCCs during tensile loading increases, the crack width decreases, and the crack morphology changes from a relatively smooth to a more tortuous pattern.
6. Partial replacement of cement with wollastonite reduces defects in the matrix, enhances matrix strength, and improves the interfacial bonding performance between PE fibers and the matrix. It optimizes the performance relationship among PE fibers, matrix, and PE fiber–matrix interface and strengthens the synergistic effect of these three components.
7. Based on the experimental results of fluidity, compressive and flexural strength, the first cracking strength, tensile strength, and tensile strain of UHTCCs, as well as the level of cement replacement with FW, should not exceed 8%, while in the case of CW it should not exceed 6%.

## Figures and Tables

**Table 1:** Chemical composition of materials.

Chemical Composition (%)	Cement	Wollastonite	Silica Sand
SiO_2_	23.89	51.62	98.20
CaO	54.83	43.48	0.30
Al_2_O_3_	8.23	0.30	0.10
Fe_2_O_3_	3.73	1.01	0.20
MgO	4.42	0.30	-
K_2_O	0.70	0.06	-
Na_2_O	0.31	0.18	-
SO_3_	3.10	0.05	-
Loss on ignition (%)	0.70	2.84	0
Total	99.91	99.84	98.80

**Table 2:** Mix proportions of UHTCC.

Number	Cement (g)	Silica Sand (g)	SP (g)	PE (g)	CW (g)	FW (g)	Water (g)
WS-0	750	250	3.75	10.3	0	0	187.5
WS-C-2	735	250	3.75	10.3	15	0	187.5
WS-C-4	720	250	3.75	10.3	30	0	187.5
WS-C-6	705	250	3.75	10.3	45	0	187.5
WS-C-8	690	250	3.75	10.3	60	0	187.5
WS-C-10	675	250	3.75	10.3	75	0	187.5
WS-F-2	735	250	3.75	10.3	0	15	187.5
WS-F-4	720	250	3.75	10.3	0	30	187.5
WS-F-6	705	250	3.75	10.3	0	45	187.5
WS-F-8	690	250	3.75	10.3	0	60	187.5
WS-F-10	675	250	3.75	10.3	0	75	187.5

**Table 3:** Influence of wollastonite on the tensile properties and microcrack of UHTCC.

Number	Initial Cracking Strength (MPa)	Tensile Strength (MPa)	Tensile Strain (%)	Number of Cracks	Average Microcrack Width (μm)
WS-0	6.4 ± 0.3	8.4 ± 0.2	3.3 ± 0.2	25 ± 3	72.0
WS-C-2	6.3 ± 0.2	8.7 ± 0.2	4.9 ± 0.1	35 ± 4	68.6
WS-C-4	6.4 ± 0.1	9.2 ± 0.1	5.1 ± 0.2	38 ± 4	67.1
WS-C-6	6.2 ± 0.2	9.5 ± 0.1	5.7 ± 0.1	46 ± 2	63.7
WS-C-8	5.8 ± 0.1	9.1 ± 0.1	4.2 ± 0.1	38 ± 3	56.6
WS-C-10	5.5 ± 0.1	8.6 ± 0.1	4.3 ± 0.1	32 ± 2	65.8
WS-F-2	6.6 ± 0.1	8.9 ± 0.1	3.8 ± 0.2	28 ± 3	69.6
WS-F-4	6.8 ± 0.1	9.4 ± 0.1	4.2 ± 0.1	30 ± 3	70.0
WS-F-6	6.7 ± 0.2	9.9 ± 0.1	4.6 ± 0.2	38 ± 2	60.8
WS-F-8	6.5 ± 0.1	10.0 ± 0.1	5.5 ± 0.1	46 ± 4	60.1
WS-F-10	5.8 ± 0.2	9.1 ± 0.1	4.6 ± 0.1	40 ± 3	59.1

## Data Availability

The original contributions presented in this study are included in the article. Further inquiries can be directed to the corresponding author.
